# Objective sleep characteristics and cognitive performances: the role of race/ethnicity

**DOI:** 10.1002/alz.089600

**Published:** 2025-01-09

**Authors:** Clémence Cavaillès, Yue Leng, Katie L Stone, Carrie B. Peltz, Kristine Yaffe

**Affiliations:** ^1^ University of California, San Francisco, San Francisco, CA USA; ^2^ Department of Psychiatry and Behavioral Sciences, University of California, San Francisco, San Francisco, CA USA; ^3^ California Pacific Medical Center Research Institute, San Francisco, CA USA; ^4^ San Francisco Veterans Affairs Health Care System, San Francisco, CA USA; ^5^ Department of Neurology, University of California, San Francisco, San Francisco, CA USA; ^6^ Department of Epidemiology, University of California, San Francisco, San Francisco, CA USA

## Abstract

**Background:**

A growing body of literature suggests that poor sleep has a negative effect on cognition. However, most prior research has predominantly used subjective sleep data and focused on non‐Hispanic White (NHW) populations. Our goal was to explore the associations between objective sleep characteristics and cognition in a racially/ethnically diverse cohort.

**Method:**

We studied 855 participants from the Health and Aging Brain Study‐Health Disparities (HABS‐HD)‐Dormir study. Objective sleep characteristics, including nighttime sleep duration, wake after sleep onset (WASO), sleep efficiency, and sleep fragmentation index, were assessed by 7‐day wrist actigraphy. Using a battery of cognitive tests, we created four cognitive composite z‐scores for executive function, attention, language, and memory. We used multivariable linear regressions to examine the associations between sleep characteristics and cognitive function, adjusting for age, race/ethnicity, sex, education, marital and cognitive status. Stratified analyses based on race/ethnicity were performed in the presence of interaction.

**Result:**

We studied 370 (43.3%) NHW, 292 (34.2%) Mexican American (MA), and 193 (22.6%) Black participants, with an average age of 66.7 (±8.4) years, and 65.1% female. Compared to NHW participants, both MA and Black participants had shorter sleep duration (7.5, 7.2, and 7.1 hours, respectively, p<0.0001), higher WASO (44.5, 57.0, 61.0 min, p<0.0001), lower sleep efficiency (91.0, 88.0, and 87.0%, p<0.0001), and higher sleep fragmentation index (24.0, 27.2, and 31.9, p<0.0001). Across the sample, longer sleep duration (per 1 hour; β = ‐0.048; p = 0.01) and higher WASO (per 30 min; β = ‐0.041; p = 0.06) were associated with worse executive function. Better sleep efficiency (per 5%; β = 0.080; p = 0.02) was associated with better executive function whereas higher sleep fragmentation index (per 10%; β = ‐0.074; p = 0.06) was associated with worse executive function in Black participants (p interaction = 0.07 and 0.008, respectively). Moreover, higher WASO (per 30 min; β = 0.075; p = 0.04) and higher sleep fragmentation index (per 10%; β = 0.072; p = 0.05) were associated with better attention function in Black participants (p interaction = 0.07 and 0.06, respectively).

**Conclusion:**

These findings indicate associations between poor sleep and both executive function and attention; and highlight evidence for racial/ethnic disparities across these relationships.

